# Management of a Multi-Room Downtime Event in a Multi-System Environment: A Case Report in Proton Therapy Operations

**DOI:** 10.1016/j.ijpt.2026.101306

**Published:** 2026-02-07

**Authors:** Perry B. Johnson, Bradlee Robbert, Trevor Fleming, Taylor Dillinger, Kevin Kirby, Kristin Heath, Nancy P. Mendenhall

**Affiliations:** 1 University of Florida Health Proton Therapy Institute, Jacksonville, FL, USA; 2 University of Florida College of Medicine, Gainesville, FL, USA

**Keywords:** Downtime, Re-planning, Operations, ProteusONE

## Abstract

**Purpose:**

To describe the management of a 5-day downtime event of a multi-room ProteusPLUS (P-PLUS) proton therapy (PT) system, including the triage and treatment of patients on a single-room ProteusONE (P-ONE) system and 2 linear accelerators.

**Methods and Materials:**

Following the failure of a radiofrequency (RF) coupler, patients were triaged for PT on the P-ONE according to medical necessity and available capacity, aiming to minimize the complexity of the P-ONE workload while ensuring each patient received at least 4 fractions of radiotherapy that week. Replanning was required across non-beam matched systems (P-ONE, TrueBeam, and Synergy) and rapidly accomplished primarily via remote planning. Treatment adaptations included extending PT availability from 12 to 19 hours, the suspension of training of students and new therapists, the delay of new starts, and the use of kV oblique imaging for brain cases in lieu of cone-beam CT (CBCT).

**Results:**

A total of 42 patients were replanned within 30 hours, with an additional 5 patients replanned thereafter. The P-ONE averaged 60 patient treatments per day during the downtime event and achieved 61 treatments in 17.5 hours on a given day. Overall, 73% of scheduled PT fractions across all systems were treated with PT, with an additional 8% treated with photon therapy (XT), 13% postponed, and 6% canceled. All patients currently under treatment received at least 4 fractions of radiation therapy, with 73.6% receiving a full 5 fractions. The case mix on the P-ONE shifted towards less complex treatments overall (+13.4% prostate).

**Conclusion:**

Swift triage, extended operating hours, and cross-platform re-planning enabled high-throughput and continuity of care during a significant downtime event. The event offers an early look at how downtime events can be successfully managed in a multi-system PT environment.

## Introduction

In August of 2025, the UF Health Proton Therapy Institute (UFHPTI) experienced a 5-day downtime event affecting the institute’s multi-room, ProteusPLUS (P-PLUS) system (Ion Beam Applications SA, Louvain-la-Neuve, Belgium). This event precipitated the transfer of several patients to the institute’s single room, ProteusONE (P-ONE) system (Ion Beam Applications SA, Louvain-la-Neuve, Belgium), which subsequently averaged 60 patient treatments per day during the event. To our knowledge, this represents the greatest number of patients treated on a P-ONE system, and importantly, was accomplished in just 17.5 hours on a given day. Ultimately, 73% of scheduled proton therapy (PT) fractions across all systems were treated with PT, with an additional 8% treated with photon therapy (XT), 13% postponed, and 6% canceled.

The management of this complex situation raises several interesting discussion points related to the timing of decisions, triage of patients, methods for replanning, changes in treatment approach, scheduling and communication with patients, communication with the PT service provider, and coordination among the treatment team. The purpose of this case report is to highlight these discussion points and describe lessons learned that may help others navigate similar events in the future. While the current arrangement at UFHPTI is somewhat unique in having 2 independent PT systems, that is, 2 cyclotrons, operating within a single building, this setup is likely to become more common in the future. This case report thus offers an early look at how downtime events can be successfully managed in a multi-system environment, or likewise how a single-room downtime event can be managed in a single-system, multi-room environment.

## Methods and materials

### Description of systems and workflows

The P-PLUS at UFHPTI includes a single cyclotron that serves 3 gantry rooms and a dedicated eye-line. Two gantry rooms have been upgraded from passive scatter to pencil-beam scanning (PBS), one with a universal nozzle and one with a dedicated nozzle. The third gantry room has not been upgraded and is currently inactive. The P-ONE includes a synchro-cyclotron that serves 1 compact gantry room. At the time of the event, a TrueBeam with an HD120 MLC (Varian Medical Systems, Palo Alto, CA) and an older Synergy (Elekta, Stockholm, Sweden) were also available for XT. Notably, none of these treatment options are beam matched, and replanning is required to move patients from one system to another. The maximum field size is also variable ranging from 30 x 40 cm on the universal nozzle of the P-PLUS to 20 x 24 cm on the P-ONE to 22 x 40 cm on the TrueBeam for MLC-defined fields. Because of this, patients receiving PT for breast cancer are primarily treated on the P-PLUS and infrequently treated on the P-ONE to avoid the need for multiple isocenters, which increases plan complexity and treatment time. In the same vein, targets with significant motion (∼1 cm) are also preferentially treated on P-PLUS’s universal nozzle due to its larger spot size in comparison to the P-ONE, which helps mitigate the interplay effect during PBS delivery. These aspects played a key role in the later triage of patients during the downtime event. On the P-ONE, cone-beam CT is commonly used for intracranial, base of skull, and head and neck cases, whereas oblique and orthogonal planar imaging is used for prostate cases. For reference, typical treatment times on the P-ONE by case type are: prostate, 12-20 minutes; intracranial or base-of-skull, 20-30 minutes; head-and-neck or breast, 25-40 minutes; and craniospinal or anesthesia, 45-60 minutes. RayStation v2023B is used for treatment planning.

### Description of event

On Friday, August 22nd, the entire P-PLUS system went offline due to a problem with the radiofrequency (RF) system. Luckily, this occurred late in the day, and only 8 patient treatments were canceled. The problem was quickly identified as a faulty RF coupler that required replacement. Initially the lead time was unclear, but by Saturday afternoon it was evident that it would take several days to source the needed part. This was discussed in text and phone communication among key leadership, including the Medical Director, Director of Physics, Technical Director, and Executive Director. A strategy was developed and refined the next morning after further discussion with the Director of Dosimetry and the lead RT manager. Re-planning was initiated early Sunday morning, and patient treatments were delivered across available systems Monday through Wednesday until the P-PLUS was restored late Wednesday evening.

### Management decisions

#### Patient triage

During the downtime, P-ONE hours were extended from 6:30 am-6:30 pm (12 hours) to 6:00 am-1:00 am (19 hours). With an optimized schedule and no interruptions, it was hypothesized that treatment of 60+ patients was possible. As the overall capacity across both PT systems was 80+ at the time of the occurrence, triage was required in order to assign patients between the P-ONE and available XT systems. Due to time constraints and the pressures of the moment, a straightforward, 2-objective strategy was adopted. The primary objective was for every patient already under treatment to receive at least 4 fractions of radiotherapy, PT or XT, that week. The secondary objective was to maximize PT treatments based on medical necessity and capacity while minimizing disruption to patients currently under treatment on the P-ONE. To meet these goals, all new starts on both the P-PLUS and P-ONE were postponed to the following week, none of which were considered urgent, current pediatric and brain cases were prioritized for PT based on medical necessity, and the remaining P-ONE capacity was filled with less complex cases that could be treated efficiently (to be further discussed in the next sections). Remaining patients were transferred to either the TrueBeam or Synergy for XT, importantly—with the expectation that if the downtime exceed 2-3 treatment days, they would be fit back within the PT schedule through rolling cancellations, again ensuring at least 4 fractions of radiotherapy were achieved for all patients.

#### Re-planning

UFHPTI employs 10 dosimetrists and uses a hybrid work model that supports remote planning. On Sunday, 8 dosimetrist worked on re-plans: 6 remotely and 2 on-site. Per institutional protocol, robust optimization with “independent beams” is normally used for PBS planning. The number of scenarios ranges from 147 for a plan with 2 beams to 7203 for a plan with 4 beams. The number of scenarios directly impacts optimization time, which can range from 35 minutes for a standard prostate case to several hours for a complicated case involving the head and neck. To accomplish faster replanning, this parameter was relaxed to “universal,” which reduced the number of scenarios to 21, and reduced optimization time to 10-60 minutes for most cases. Recent correspondence with several PT centers indicates that the use of independent beams is not widespread and generally considered very conservative.^1^ Additionally, robust evaluations of multiple cases showed little impact on plan dosimetry, an example of which is highlighted in Supplementary Material Figures S1-S3. Thus, this tradeoff was deemed acceptable under the circumstances of the moment.

#### Patient treatment

Each radiation therapist worked 10 hours either during an early, mid, or late shift, which was their normal schedule except for changes in when each shift began. On the P-ONE, 3 therapists managed patient treatments, a fourth proactively retrieved patients from the lobby, and at least 2 others reviewed charts, managed the schedule, and communicated with patients. Training of junior therapists and students was also suspended during the downtime event. For brain cases transferred to the P-ONE, kV oblique imaging was used for alignment in lieu of cone-beam CT. No other changes were made to imaging or setup protocols.

For patients treated with XT, if insurance required a new authorization for change in modality, this was secured for up to 5 fractions (sometimes retrospectively), and the treatments were billed as XT. If clinical documentation was required, a standard letter was provided that described the machine downtime and need for XT to avoid treatment delays. Re-authorization was successfully achieved for all but 2 patients whose insurance provider, UnitedHealthcare, did not accept retrospective authorizations.

## Results

Table 1 shows the number of patients initially scheduled across both the P-PLUS and P-ONE systems and how these patients were managed throughout the week. At the time, the P-PLUS was scheduled to carry two-thirds of the PT load, which is proportional to the percentage of active gantry rooms represented by that system. From this starting point, 42 patients were replanned. Of these, 34 were transferred to the P-ONE, 6 were transferred to the TrueBeam, and 2 were transferred to the Synergy. This was accomplished in approximately 30 hours of planning, Sunday morning to Monday afternoon. Five more patients were re-planned and added to the clinical schedule for Tuesday. Across the event, only 10 patients changed modalities and received XT: 6 for breast/chest-wall cancer, 2 for sites with significant motion (lung and pancreas), 1 for a lower neck/shoulder nerve sheath tumor, and 1 for treatment of the larynx, for which a TrueBeam plan already existed.

**Table 1:** Patient management of ProteusPLUS (P-PLUS) patients across the ProteusONE (P-ONE) and available linear accelerators on the Monday, Tuesday, and Wednesday of the downtime event.

	Scheduled			Postponed		Canceled	Transferred			Continued	Time (hr:min)
	Total PT	P-PLUS	P-ONE	P-PLUS	P-ONE	P-PLUS	Synergy	TrueBeam	P-ONE	P-ONE	
Monday	84	59 (70%)	25 (30%)	8	2	9	2	6	34	23	18:45
Tuesday	82	56 (68%)	26 (32%)	7	3	2	2	7	38	23	18:31
Wednesday	78	53 (68%)	25 (32%)	8	3	3	1	2	39	22	17:31
	Scheduled			Postponed		Canceled	XT		PT		
Total	244			31 (13%)		14 (6%)	20 (8%)		179 (73%)		

Among all patients currently receiving PT, 77% were treated with PT on Monday, 85% on Tuesday, and 91% on Wednesday (Table 2). All patients received at least 4 fractions of radiation therapy, with 73.6% receiving a full 5 fractions. Despite treating fewer patients on Monday (57) than on Wednesday (61), treatment time was longest on Monday (18 hours, 45 minutes) and shortest on Wednesday (17 hours, 31 minutes). This was primarily due to more imaging on Monday during the initial setup of patients transferred to the P-ONE. Notably, 4 pediatric anesthesia cases were treated during this timeframe, requiring a total of 2.5 hours of machine time per day.

**Table 2:** Daily percentage of the initially scheduled and current proton therapy patient volumes, including the ProteusPLUS (P-PLUS) and/or ProteusONE (P-ONE), treated on the P-ONE.

Patient volume	Treated on P-ONE		
	Monday	Tuesday	Wednesday
Scheduled P-PLUS	58%	68%	74%
Scheduled P-PLUS & P-ONE	68%	74%	78%
Current P-PLUS	67%	78%	87%
Current P-PLUS & P-ONE	77%	85%	91%

**Figure 1:**
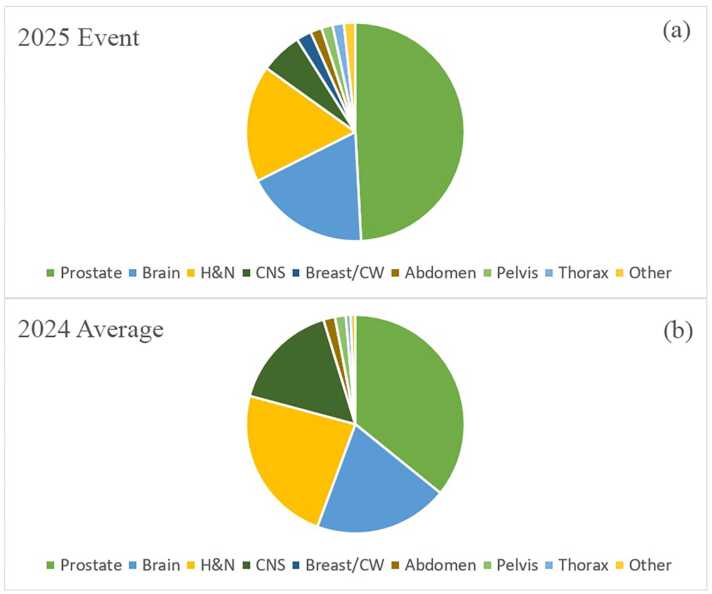
Top panel (a), case mix on ProteusONE (P-ONE) during the downtime event; Bottom panel (b), average case mix on the P-ONE during 2024.

Figure 1a highlights the case mix treated on the P-ONE during the downtime event. The majority of patients were treated for disease of the prostate (49.2%), followed by the brain (18.4%), head and neck (17.3%), and CNS (6.1%). For comparison, the average case mix on the P-ONE during 2024 is shown in Figure 1b and highlights the differential between the 2 time periods: prostate (+13.4%), brain (−1.4%), head and neck (−6.2%), CNS (−10.0%).

## Discussion

Given the complexity of PT and XT delivery systems, occasional downtime is expected. The successful management of these situations begins with clear communication with the service provider to best understand the nature of the problem and the likelihood of recovery within a specific timeframe. To highlight how timing and uncertainty play a role, Table 3 summarizes the text exchange between the service provider’s site manager and UFHPTI leadership, along with the corresponding response. Because the downtime began on a weekend, the team had time to gather more information before taking action. Once it was certain that Monday’s treatments would be affected and that the replacement coupler had not yet been sourced, both the service provider and the clinical team escalated their response. Treating current P-ONE patients that Sunday was initially considered in order to free capacity during the week. However, difficulty arranging coverage at the last minute (including nursing, therapists, physicians, physics, front desk, security, etc.) led to this plan being abandoned. By Sunday morning, the picture was clear, and the action plan described previously was initiated.

**Table 3:** Summary of the text exchange between the service provider’s site manager and UFHPTI leadership, along with the corresponding response.

Day	Time	Summary of text thread	Response (3 treatment days affected)
Friday	5:42 pm	RF failure, details pending	Wait for more information
Saturday	8:12 am	RF coupler needs replacement, availability of part unknown, Monday treatment in jeopardy, situation escalated regionally	Wait for more information
Saturday	12:12 pm	Still searching for part, situation escalated globally	Wait for more information
Saturday	4:38 pm	No spare part in US, still searching globally, Monday treatment unlikely	Select leadership begins to develop action plan to triage patients for PT and XT
Saturday	7:28 pm	Still searching globally, Tuesday treatment also unlikely	Action plan discussed more broadly within leadership team
Sunday	9:42 am	Part identified outside US, full recovery not expected until at least Thursday	Action plan finalized and re-planning of all patients started, action plan communicated to providers and relevant personnel

To further analyze this scenario, Table 4 presents a hypothetical response had the event begun Sunday rather than Friday. In that case, the risk of at least 1 missed treatment day would have been high from the start, and replanning would have begun much sooner, with priority given to pediatric patients and those scheduled to receive their final fraction (knowing that many patients arrange travel around their completion date and because finishing a patient’s treatment frees up a daily slot for someone else). As illustrated here, when the recovery timeframe is uncertain, a tiered approach is utilized, and escalation or de-escalation occurs as the situation becomes more defined. As a further comment, treating on the following Saturday would have been strongly considered had all patients not received at least 4 treatments during the week.

**Table 4:** Same as Table 3 but showing a hypothetical response had the event occurred on a Sunday rather than Friday, affecting 2 more treatment days.

Day	Time	Summary of text thread	Response (5 treatment days affected)
Sunday	5:42 pm	RF failure, details pending	Wait for more information
Monday	8:12 am	RF coupler needs replacement, availability of part unknown, Tuesday treatment in jeopardy, situation escalated regionally	Start re-planning select patients (pediatric, brain, final treatments), postpone new starts
Monday	12:12 pm	Still searching for part, situation escalated globally	Develop action plan for all patients and initiate plan
Monday	4:38 pm	No spare part in US, still searching globally, Tuesday/Wednesday treatment unlikely	Attempt to treat select patients on ProteusONE
Monday	7:28 pm	Still searching globally, Thursday treatment also unlikely	Continue re-planning all patients for PT and XT
Tuesday	9:42 am	Part identified outside US, full recovery not expected until at least Saturday	Provide PT or XT for all patients, plan to provide 4th fraction on Saturday as needed

For treatment planning, the creation of upfront backup plans for a subset of patients is a strategy that has been implemented at other facilities and previously considered at UFHPTI.^2^ Two developments—the increase in computing power (and thus faster calculation) and the ability to plan remotely—have shifted the discussion away from this approach. Re-planning 42 patients within 30 hours demonstrates that even very difficult situations can be managed effectively when an experienced team has sufficient access to plan and verify cases promptly; such access is now more widespread than ever via remote connections. Additionally, considering a future where an institution may operate multiple PT systems that are beam-matched, replanning would not be necessary, further simplifying the workflow. Re-planning was eased in the current situation by the use of a single treatment planning system for both proton and photon planning. This is not always the case in the PT community, and the use of modality-specific treatment planning systems would require additional effort to transfer data for patients moving from PT to XT.

Regarding patient throughput, the ability to treat 60+ patients on the P-ONE can be attributed to a number of factors, including 1) a simplified case mix (i.e., more prostate and brain cases), 2) the stability of the P-ONE that week, which operated with minimal interruptions during the event, 3) the inherent efficiency of the P-ONE when relying heavily on kV oblique imaging, and 4) the experience level and proficiency of the radiation therapists who worked closely as a team to manage the workload. Notably, extensive cross-training across modalities and systems enabled therapists to adapt quickly and provide additional support to available systems as needed. Dosimetrists and physicists, who routinely plan and verify treatments across modalities, likewise were able to fully contribute throughout the downtime event. While a daily workload of ∼60 patients was maintained for 3 days, several factors would challenge this capacity over longer timeframes, including a lack of flexibility in the schedule to absorb even minor delays, limitations on the case mix, risk of staff burnout from extended hours, and a likely increase in machine downtime with prolonged use.

One of the more challenging issues throughout the event was patient triage—both how decisions were made and how they were communicated to patients. Several factors can influence these decisions: medical necessity (e.g., prioritization of pediatric patients), equity (considering patients on both the down and available systems), operational and technical factors, individual circumstances such as patient travel, finances, and mental state, and the concerns of the business operation. As mentioned previously, there was a strong desire to act decisively while keep the strategy simple, since the schedule could not afford unexpected delays or additional lengthy procedures. Ergo, when operating at maximum capacity, simplicity likely offers the greatest margin for success. For example, UFHPTI rarely treats breast cases on the P-ONE, and adding a new, complex workflow would have required a certain level of flexibility that wasn’t available at the time. The team also chose XT for the 2 patients with significant target motion to avoid a more complex treatment on the P-ONE, which would have involved multiple fields and repainting. Finally, one patient already had a TrueBeam plan available, so transferring them immediately was the most straightforward path that allowed treatment to resume as soon as possible. For these patients, it was believed that a few fractions of XT could sustain continuity of care without compromising clinical objectives. The leadership team made these decisions and communicated them to all providers by noon on Sunday, noting that individual exceptions could be considered but that flexibility was limited. Providers are best positioned to know the needs of their patients and advocate for them if truly necessary, and as a result, one patient who was initially slated for XT was moved back to PT.

In terms of communicating schedule changes to patients, this was mostly handled by the therapists. However, when treatments were postponed or moved to XT, patients clearly preferred to speak directly with a provider. This happened on a number of occasions, and in hindsight, it would have been more efficient to have providers manage these specific conversations from the outset, recognizing that this may not always be an available option.

Ultimately, how patient triage is performed will depend on the dynamics and minutiae of each facility and the circumstances surrounding an individual event. At UFHPTI, prior experience has informed the general strategy described above, but codifying this as a formal policy is a future action item identified during a debriefing of the event. As this report demonstrates (and as the saying goes), “there are no solutions, only tradeoffs.”^3^ Thus, flexibility and patience are paramount, and reaching consensus among providers and stakeholders on triage procedures before an event occurs would likely make implementation less difficult. Finally, for situations like these, it is also critical to plan for the impact to staff beyond the immediate treatment team—nursing, front desk, security, and others—since extended hours and potential treatment on Saturday or Sunday requires their support. A successful outcome thus depends on coordination across all aspects of a facility. A 10-point summary of “lessons learned” from this event is provided in the Supplementary Material.

## Conclusion

An RF failure of the P-PLUS system prompted rapid, coordinated re-planning, with treatments shifted to the facility’s P-ONE and available linear accelerators. Patient triage was guided by medical necessity and available capacity, aiming to minimize the complexity of the P-ONE workload while ensuring each patient received at least 4 fractions of radiotherapy that week. Operational adaptations included extended treatment hours, suspension of training, and the use of kV oblique imaging for brain cases. The case mix on the P-ONE also shifted towards less complex treatments. These changes, as implemented via a highly experienced therapy team, made it possible to treat 60+ patients per day on the P-ONE system, providing continuation of PT for the vast majority of affected patients.

## Research Support

This research received no external financial or non-financial support.

## Relationships

There are no additional relationships to disclose.

## Patents and Intellectual Property

There are no patents to disclose.

## Other Activities

There are no additional activities to disclose.

## CRediT authorship contribution statement

Perry B. Johnson: Conceptualization, data curation, formal analysis, writing – original draft. Bradlee Robbert: Methodology, data curation, formal analysis, writing – review and editing. Trevor Fleming: Methodology, formal analysis, writing – review and editing. Taylor Dillinger: Methodology, writing – review and editing. Kevin Kirby: Methodology, writing – review and editing. Kristin Heath: Methodology, writing – review and editing. Nancy P. Mendenhall: Methodology, writing – review and editing.

## Declaration of Conflicts of Interest

The authors declare the following financial interests/personal relationships, which may be considered as potential competing interests: Two authors of this manuscript serve in editorial capacities for IJPT, Nancy Mendenhall as Editor-in-Chief and Perry Johnson as Associate Editor. Given their roles, neither were involved in the peer review of this article and had no access to information regarding its peer review. Full responsibility for the editorial process for this article was delegated to another journal editor. If there are other authors, they declare that they have no known competing financial interests or personal relationships that could have appeared to influence the work reported in this paper.

## Declaration of Generative AI and AI-Assisted Technologies in the Writing process

During the preparation of this work, the author(s) used gpt-5 in order to provide minor editing and suggestions of alternative phrasing. After using this tool/service, the author(s) reviewed and edited the content as needed and take(s) full responsibility for the content of the published article.

## Footnotes

Appendix A Supplementary data associated with this article can be found in the online version at doi:10.1016/j.ijpt.2026.101306.
