# Supplementary material for: Management of a Multi-Room Downtime Event in a Multi-System Environment: A Case Report in Proton Therapy Operations
Source: Int J Part Ther. 2026 Feb 7;19:101306. doi: 10.1016/j.ijpt.2026.101306 (PMC12925582; doi:10.1016/j.ijpt.2026.101306)
Supplement: Supplementary file 1 — Supplementary material [file mmc1.docx]

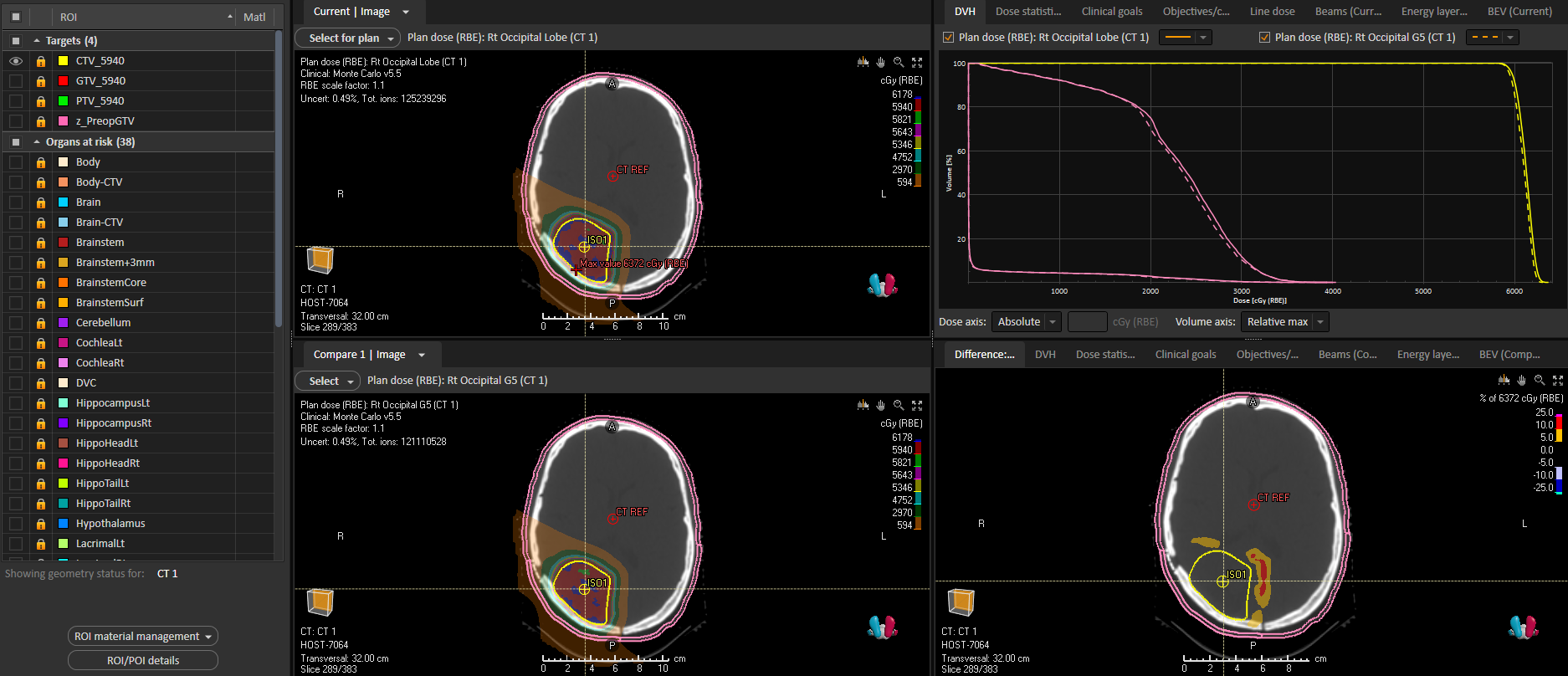


**Figure S1.** Isodose plots, dose volume histogram, and dose difference for the original, ProteusPLUS treatment plan (top-middle panel) and the modified, ProteusONE treatment plant (bottom-middle panel). Notably, the spot sizes of the dedicated nozzles of the ProteusPLUS (Gantry Room 1) and the ProteusONE are very similar, and as expected, the plan dosimetry is also very similar when using an identical field arrangement.


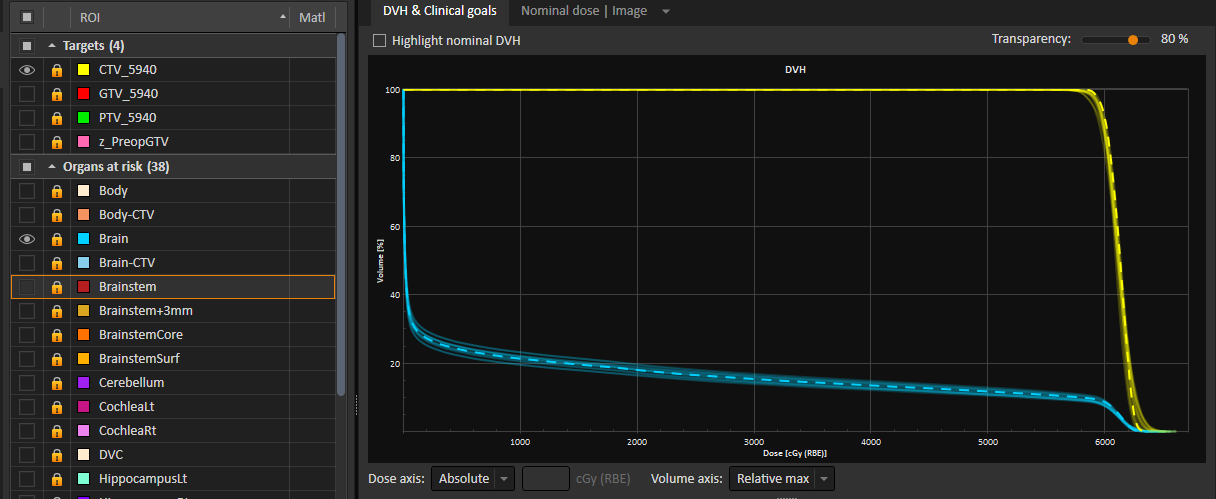


Original plan, 3 mm / 4% range uncertainty


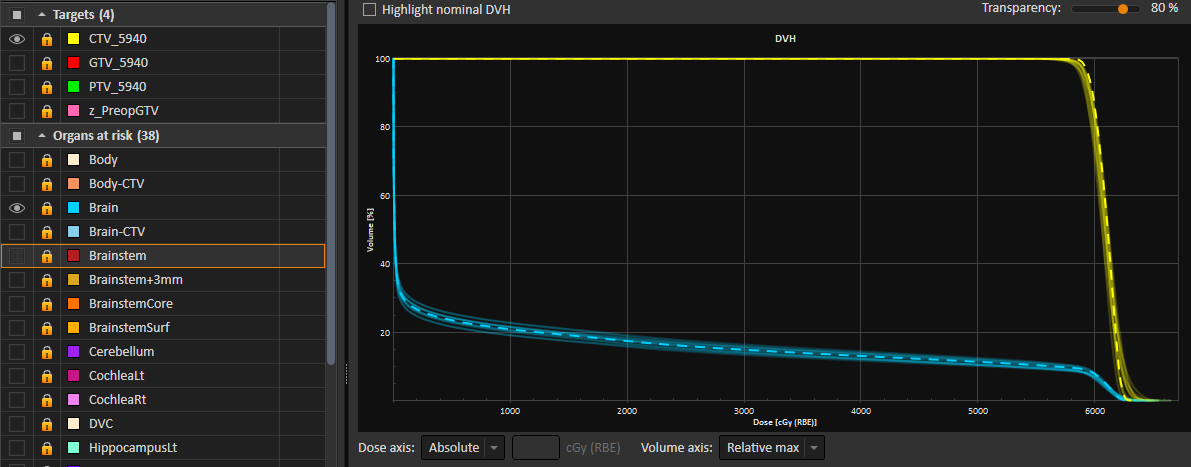


Modified plan, 3 mm / 4% range uncertainty

**Lessons Learned**

- Ensure clear, early communication with the service provider; use a tiered response when recovery time is uncertain, and consider weekend treatments if necessary to ensure continuity of care.
- Be prepared to obtain or retrospectively secure insurance authorizations when the modality changes, noting that some payers may not accept retrospective approvals.
- Cross-training is extremely valuable as it allows reassignment of staff to available resources ensuring maximum effort can be applied throughout a prolonged downtime event.
- Rapid replanning is feasible via remote work and can mitigate the need for upfront backup plans; using a single TPS for both PT and XT and/or having beam-matched systems will further simplify the workflow.
- Temporarily relax highly conservative planning parameters (e.g., “independent beams” to “universal”) to speed optimization when dosimetry impact is minimal and time is critical.
- Have providers be available as necessary to handle sensitive patient communications (postponements or modality changes) to reduce redundant communication and provide a better patient experience.
- When operating at maximum capacity, simplicity likely offers the greatest margin for success.
- Involve ancillary staff in the overall response plan—nursing, front desk, security, and others—as extended hours and potential weekend treatments will require their support.
- Seek consensus on patient triage before an event occurs; codify downtime management into policy, and embrace that there are tradeoffs rather than perfect solutions.
- With an optimized case mix, an experienced team, and a little luck, a single-room P-ONE can temporarily handle a very high patient volume.
